# Risk Factors of Superimposed Preeclampsia in Women with Essential Chronic Hypertension Treated before Pregnancy

**DOI:** 10.1371/journal.pone.0062140

**Published:** 2013-05-06

**Authors:** Edouard Lecarpentier, Vassilis Tsatsaris, François Goffinet, Dominique Cabrol, Baha Sibai, Bassam Haddad

**Affiliations:** 1 University Paris 12, CHI Creteil, Creteil, France; 2 University Paris 5, AP-HP Cochin Port-Royal, Paris, France; 3 University of Cincinnati, Cincinnati, Ohio, United States of America; 4 PREMUP Foundation, Paris, France; 5 CRC CHI Creteil, Creteil, France; College of Pharmacy, University of Florida, United States of America

## Abstract

**Objective:**

To determine risk factors of superimposed preeclampsia in women with essential chronic hypertension receiving antihypertensive therapy prior to conception.

**Methods:**

A retrospective study of 211 patients that analyzed risk factors of superimposed preeclampsia at first prenatal visit. Variables with a p<.1 at univariate analysis were included in a logistic regression analysis. P<.05 was considered as significant.

**Results:**

Superimposed preeclampsia occurred in 49 (23.2%) women. In logistic regression analysis, previous preeclampsia [OR: 4.05 (1.61–10.16)], and mean arterial blood pressure of 95 mmHg or higher [OR: 4.60 (1.94–10.93)] were associated with increased risk of superimposed preeclampsia. When both variables were present, sensitivity, specificity, positive predictive value, negative predictive value, and likelihood ratio for superimposed preeclampsia were 43%, 94%, 70%, 85%, and 7.71 (95% CI: 3.20–18.57), respectively.

**Conclusion:**

In essential chronic hypertensive women, previous preeclampsia and mean arterial blood pressure of 95 mmHg or higher are associated with increased risks of superimposed preeclampsia.

## Introduction

Chronic hypertension is a relatively common disorder occurring in approximately 1–5% of pregnant women; rates depending on the population studied and the criteria used for the diagnosis [1]. Because of increasing maternal age, obesity, and type 2 diabetes worldwide, it is expected that the prevalence of chronic hypertension in pregnancy will continue to increase. The study ENNS (National Nutrition Health Survey) cross-sectional survey in France between 2006 and 2007 reveals a prevalence of chronic hypertension of 4.1% in women between 18 and 34 years and of 8.3% between 35 and 44 years. Hypertension was known to the patient in 22.3% of cases between 18 and 34 years and in 55.5% of cases between 35 and 44 years [2]. Pregnancies complicated by chronic hypertension are at increased risk of superimposed preeclampsia, abruptio placenta, fetal growth restriction, preterm delivery, and perinatal death [1], [3]–[8].

In women with chronic hypertension, the risk of superimposed preeclampsia is increased in black ethnic origin, raised body mass index (BMI), smoking, booking systolic blood pressure of 130 to 139 mm Hg, and diastolic blood pressure of 80 to 89 mm Hg [3], and in women with chronic hypertension ≥4 years [7]. Conflicting results have been published on the relationship between a history of preeclampsia and the rate of superimposed preeclampsia in subsequent pregnancies [3], [4], [7]. These differences might be related to inclusion of heterogeneous population of women with hypertension; in some studies women had only essential hypertension whereas in others women had all forms of hypertension. In addition, most of the studies included women who were diagnosed with chronic hypertension on the basis of either having hypertension prior to pregnancy or during the first 20 weeks gestation. Moreover, none of the studies reported to date have included only women who received antihypertensive medication prior to conception.

The objective of this study was to identify risk factors for superimposed preeclampsia at first prenatal visit in women with essential chronic hypertension receiving antihypertensive medication prior to conception.

## Methods

This retrospective study included women with chronic hypertension delivered between 1 January 2004 and 31 December 2007 who were identified from the hospital computer databases of two university hospital centers (CHI Creteil and AP-HP Cochin Port-Royal Paris). Every medical chart was reviewed to collect the data. The criteria used to select women with chronic hypertension was a diagnosis of hypertension that needed a treatment before the onset of the pregnancy. Exclusion criteria were: women with multiple pregnancies, women with secondary hypertension, women with proteinuria at less than 20 weeks’ gestation, women considered as having a chronic hypertension but without any treatment at first prenatal visit, women transferred from other maternities, pregnancies complicated by fetal malformations.

The data collected from medical records included: age, pre-pregnancy BMI, parity, ethnic origin, tobacco use during pregnancy, duration of hypertension, past obstetric history, antihypertensive treatment, treatment with low dose aspirin, maternal systolic and diastolic blood pressure at booking, presence or absence of proteinuria at first prenatal visit, maternal and neonatal outcomes. The blood pressure was obtained with automated device, patient in sitting up position. The mean arterial pressure was calculated from brachial systolic and diastolic blood pressure (BP), according to the following formula: Mean arterial pressure = [systolic BP+(2*diastolic BP)]/3. Superimposed preeclampsia was defined as a new onset proteinuria (0.3 g of protein or more in a 24-hour specimen) after 20 weeks’ gestation and without proteinuria early in pregnancy (less than 20 weeks’ gestation). Fetal growth restriction (FGR) was defined as a birth weight <5th percentile [9]. Abruptio placenta was diagnosed according to clinical findings and/or placental examination. HELLP syndrome (hemolysis, elevated liver enzymes, low platelet count) was defined according to Sibai’s criteria [10].

We analyzed the risk factors that may influence the rates of superimposed preeclampsia. Categorical variables are presented as percentage, and continuous variables as mean and SD. Categorical variables were compared with *X^2^* square or Fisher’s exact test and continuous variables with a two-tailed student *t* test. Variables with a p<.1 were included in a logistic regression analysis. Data are expressed as odds ratio (OR) with 95% confidence interval (CI). P<.05 was considered as significant. Positive likelihood ratio with 95% CI was calculated for the prediction analysis. The statistical software Statview 5.0 (SAS Institute) was used for statistical analysis, and REALbasic (2002) for Receiver Operative Characteristics (ROC) analysis.

The evaluation of chronic hypertensive women did not need approval of our ethical committee because it was a retrospective analysis of data in women who received standard management at both hospitals. Therefore, our ethical committee has waved requirement for approval.

## Results

The study population consisted of 362 women with chronic hypertension. Women transferred from other centers (n = 61), women with secondary hypertension or with chronic hypertension and pre-existing proteinuria before 20 weeks’ gestation (n = 20), women with fetal anomalies (n = 4), and women not receiving antihypertensive medication prior to pregnancy (n = 66) were excluded. The analysis concerned exclusively the 211 women with essential chronic hypertension who were receiving antihypertensive therapy prior to conception.

Table 1 describes maternal characteristics at booking (first visit) and Table 2 summarizes overall maternal and perinatal outcomes. No maternal death occurred among the women.

**Table 1:** Characteristics of 211 women with essential chronic hypertension.

Maternal age (y, mean ± SD)	35.7±4.8
Pre-pregnancy BMI (Kg/m^2^, mean ± SD)(n = 202)	27.9±6.5
Ethnic origin Black # (%)	113 (53.6)
Ethnic origin White # (%)	94 (45.5)
Ethnic origin Other # (%)	4 (1.9)
Nulliparous # (%)	39 (18.5)
Previous preeclampsia # (%)	45/172 (26.1)
Previous FGR # (%)	38/172 (22.1)
Previous abruptio placenta # (%)	9/172 (5.2)
Booking gestational age (wk, mean ± SD)	18.1±5.6
Tobacco use # (%)	20 (9.5)
Booking systolic blood pressure(mmHg, mean ± SD)	131.3±14.8
Booking diastolic blood pressure(mmHg, mean ± SD)	78.2±11.8
Booking MAP (mmHg, mean ± SD)	95.9±11.7
Duration of hypertension (y, mean ± SD)	4.6±3.5
Antihypertensive medication at booking	
One drug only # (%)	152 (73.4)
Two drugs # (%)	45 (21.7)
More than two drugs # (%)	10 (4.8)
Antihypertensives withdrawn duringpregnancy # (%)	17 (8.1)
Aspirin use # (%)	49 (23.2)
Gestational diabetes # (%)	41 (19.4)

FGR: fetal growth restriction; MAP: mean arterial blood pressure.

**Table 2:** Maternal and perinatal outcomes of 211 women with essential chronic hypertension.

Superimposed preeclampsia-HELLP # (%)	49 (23.2)
Preeclampsia <34 # (%)	19 (9)
Eclampsia # (%)	2 (.9)
Abruptio placenta # (%)	4 (1.9)
Mean gestational age atdelivery ± SD	36.9±4.1
<37 weeks # (%)	61 (28.9)
<34 weeks # (%)*	39 (18.5)
<32 weeks # (%)	29 (13.7)
Birth weight (g, mean ± SD)	2690±971
<5th centile # (%)	37 (17.5)
Umbilical arterial blood pH(mean ± SD)	7.26±0.11
1 min Apgar score <7 # (%)	30/204 (14.7)
5 min Apgar score <7 # (%)	9/204 (4.4)
Admission to neonatal care # (%)	52/204 (25.5)
Perinatal loss # (%)†	8 (3.8)

* Causes of delivery <34 wks: 19 for PE, and 20 for other reasons (12 abnormal fetal heart rate monitoring with FGR, 1 abruption, 4 pregnancy terminations for fetal death, 2 preterm premature rupture of membranes, and 1 pregnancy termination for very severe FGR at 28 weeks’ gestation with birth weight at 450 g).

† 5 fetal deaths, 2 fetal terminations [1 for very severe FGR (see above) and 1 for very early onset of severe preeclampsia at 22 weeks’ gestation], and 1 neonatal death.

The rate of superimposed preeclampsia was 23.2%. All women with HELLP syndrome had preeclampsia. Table 3 shows perinatal outcomes according to the occurrence of superimposed preeclampsia. The following variables obtained at first prenatal visit: black ethnicity, nulliparity, previous preeclampsia, previous FGR, systolic and diastolic blood pressure at booking, mean arterial blood pressure ≥95 mmHg at booking, and duration of hypertension ≥4 years were significantly associated with the occurrence of superimposed preeclampsia in univariate analysis (Table 4). In contrast, the rate of superimposed preeclampsia was not affected by maternal age, BMI, tobacco use during pregnancy, antihypertensive medication (one drug only versus several drugs), and aspirin treatment.

**Table 3:** Perinatal outcomes according to the occurrence of superimposed preeclampsia.

	PE (n = 49)	No PE(n = 162)	p
Mean gestational age atdelivery ± SD	34.0±4.7	37.5±3.6	<.05
<37 weeks # (%)	28 (57.1)	32 (19.8)	<.05
<34 weeks # (%)	19 (38.8)	20 (12.3)	<.05
<32 weeks # (%)	14 (28.6)	15 (9.3)	<.05
Birth weight (g, mean ± SD)	2105±1032	2864±884	<.05
<5th centile # (%)	9 (18.4)	28 (17.3)	NS
Abruptio placenta # (%)	3 (6.1)	1 (.6)	<.05
Umbilical arterial blood pH(mean ± SD)	7.24±0.14	7.27±0.1	NS
1 min Apgar score <7 # (%)	9/47 (19.1)	20/157 (12.7)	NS
5 min Apgar score <7 # (%)	5/47 (10,6)	5/157 (3.2)	NS
Admission to neonatal care # (%)	25 (51)	27 (16.7)	<.0001
Perinatal loss # (%)	2 (4.1)	6 (3.7)	NS

PE: superimposed preeclampsia.

**Table 4:** Factors associated with development of superimposed preeclampsia by univariate and multivariate* analysis at first prenatal visit.

Characteristics	PE (n = 49)	No PE (n = 162)	p	Multivariate analysis OR(95% CI)	P
Pre-pregnancy BMI (Kg/m^2^, mean ± SD)	n = 47 27.6±5.5	n = 155 28±6.8	.68		
Booking gestational age (wk, mean ± SD)	18.3±5.5	18±5.6	.75		
Maternal age (years)			.35		
<30 # (%)	7 (14.3)	15 (9.3)			
30–35 # (%)	18 (36.7)	50 (30.9)			
≥35 # (%)	24 (49)	97 (59.8)			
Ethnicity			.005		
White # (%)	13 (26.5)	81 (50)		1	ref
Black # (%)	36 (73.5)	77 (47.5)		1.65 (0.73–3.72)	.22
Other # (%)	0	4 (2.5)			
Nulliparous # (%)	4 (8.1)	35 (21.6)	.03	0.70 (0.20–2.37)	.56
Previous preeclampsia # (%)	24/45 (53.3)	21/127 (16.5)	<.0001	4.05 (1.61–10.16)	.003
Previous FGR # (%)	14/45 (31.1)	24/127 (18.9)	.03	0.99 (0.36–2.69)	.98
Previous abruptio placenta # (%)	4/45 (8.9)	5/127 (3.9)	.21		
Tobacco use # (%)	2 (4.1)	18 (11.1)	.82		
Booking systolic blood pressure (mmHg)			.005		
<130 # (%)	11 (22.4)	76 (46.9)			
130–140 # (%)	16 (32.7)	45 (27.8)			
≥140 # (%)	22 (44.9)	41 (25.3)			
Booking diastolic blood pressure (mmHg)			.0004		
<80 # (%)	14 (28.6)	94 (58)			
80–90 # (%)	21 (42.8)	50 (30.9)			
≥90 # (%)	14 (28.6)	18 (11.1)			
Booking MAP≥95 mmHg # (%)	40 (81.6)	73 (45.1)	<.0001	4.60 (1.94–10.93)	.0006
Duration of hypertension ≥4 years # (%)	34 (69.4)	69/152 (45.4)	.003	2.09 (0.95–4.60)	.07
Antihypertensive at booking	n = 48	n = 159	.86		
One drug only # (%)	35 (72.9)	117 (73.6)			
Two drugs # (%)	10 (20.8)	35 (22)			
> two drugs # (%)	3 (6.3)	7 (4.4)			
Aspirin use # (%)	15 (30.6)	34 (21)	.16		

* logistic regression analysis performed for variables with a p<.1 at univariate analysis at booking; PE: superimposed preeclampsia.

We have chosen to include mean arterial blood pressure ≥95 mmHg at multivariate analysis and to analyze its predictive status, rather than systolic or diastolic blood pressure, because mean arterial blood pressure had the best prediction analysis for superimposed preeclampsia [area under curve at ROC analysis of.72 (p<.0001) for mean arterial blood pressure, .69 (p<.0001) for diastolic blood pressure, and.68 (p = .0001) for systolic blood pressure] and 95 mmHg was the best cut-off for sensitivity and specificity. Multivariate analysis showed that previous preeclampsia, and mean arterial blood pressure ≥95 mmHg were independently associated with the occurrence of superimposed preeclampsia (Table 4).

We analyzed the prediction for the occurrence of superimposed preeclampsia using the variables that were selected in logistic regression for superimposed preeclampsia at first prenatal visit: previous preeclampsia and mean arterial blood pressure. The ROC analysis of mean arterial blood pressure and previous preeclampsia showed an area under curve at of.77 (p<.0001). The prediction analysis of previous preeclampsia and MAP≥95 mmHg is shown in Table 5. When both variables were present, sensitivity, specificity, positive predictive value, negative predictive value, and likelihood ratio for superimposed preeclampsia were 43%, 94%, 70%, 85%, and 7.71 (95% CI: 3.20–18.57), respectively.

**Table 5:** Prediction for the occurrence of superimposed preeclampsia according to significant variables selected by logistic regression analysis at booking.

	SE %	SP %	PPV %	NPV%	Positive LR (95% CI)
Previous preeclampsia (1)	49	87	53	85	3.76 (1.82–7.75)
MAP≥95 mmHg (2)	82	55	35	91	1.81 (0.83–3.98)
Composite variable1or 2	88	47	33	93	1.65 (0.67–4.10)
Composite variable 1and 2	43	94	70	85	7.71 (3.20–18.57)

SE: sensitivity, SP: specificity, PPV: positive predictive value, NPV: negative predictive value, LR: likelihood ratio.

Composite variable 1and 2: all both are present: previous preeclampsia, MAP≥95 mmHg.

Composite variable 1or2: at least one of: previous preeclampsia, MAP≥95 mmHg.

Of the 49 patients with superimposed preeclampsia, 19 occurred at less than 34 weeks’ gestation. Of all variables analyzed, only SBP at booking was associated with increased risk of early onset compared to late onset of preeclampsia (SBP≥140 mmHg: 74% vs. 27%, SBP 130–140 mmHg: 21% vs. 40%, and SBP<130∶5% vs. 33%, respectively, p = .004).

## Discussion

The main goal of this study was to analyze factors obtained in the first half of the pregnancy that may be associated with superimposed preeclampsia in women with essential chronic hypertension that needed a treatment before the onset of pregnancy. In contrast to other studies [3]–[7], we have focused on essential chronic hypertension women receiving antihypertensive therapy prior to pregnancy for two reasons: 1- the diagnosis of chronic hypertension was already made, 2- the results are not biased by the primary disease as it may be observed in secondary chronic hypertension related to nephropathy or auto-immune disease. In addition, only two studies have focused on women with chronic essential hypertension, one of these studies did not specify whether or not patients were treated by antihypertensives [6], and in the second one almost half of the women had no antihypertensives before the onset of pregnancy [4].

The main findings of the study were: 1- superimposed preeclampsia occurred in 23.2% of women, 2- previous preeclampsia, and mean arterial blood pressure ≥95 mmHg at booking was associated with increased risk of superimposed preeclampsia.

The rate of superimposed preeclampsia of 23.2% observed in our study is in agreement with that reported in other studies, ranging from 17% to 34.9% [3]–[8].

In several of these studies, conflicting results concerning the relationship between a history of previous preeclampsia and the recurrence of preeclampsia in a subsequent pregnancy have been reported. Sibai et al. showed that the risk of superimposed preeclampsia was greater in chronic hypertensive women who had preeclampsia during a previous pregnancy (32% vs. 23%; OR: 1.6; 95% CI: 1.1–2.3) [7], as well as Chappell et al. (OR = 1.95, 95% CI: 1.25–3.04) [3]. A recent study, however, reported that in women with chronic hypertension a history of preeclampsia was not associated with increased risk of superimposed preeclampsia (OR = 1.28, 95% CI: 0.78 to 2.1) [4]. This latter study, however, had only 61% power to detect differences in rate of superimposed preeclampsia between groups [4]. In our study, among the women who developed preeclampsia 53.3% had a history of preeclampsia in a previous pregnancy, and previous preeclampsia was clearly associated with superimposed preeclampsia in subsequent pregnancy in the logistic regression analysis (Table 4). Along with others, blood pressure at first prenatal visit (systolic, diastolic, and mean arterial) was associated with increased risk of superimposed preeclampsia at univariate analysis [3]. The prediction of a history of preeclampsia, and mean arterial blood pressure ≥95 mmHg for superimposed preeclampsia in our population were encouraging, particularly, when both variables were present at first prenatal visit. Although sensitivity was only of 43%, positive predictive value of 70% was extremely high, and the likelihood ratio to have a superimposed preeclampsia was 7.71-fold increase. These two variables simply obtained early during the pregnancy may help to select women at very high risk of superimposed preeclampsia that might be eligible for low dose aspirin treatment. Although the usefulness of low dose aspirin in the prevention of superimposed preeclampsia is not established in women with chronic hypertension [11]–[12], this may be related to the late start (after 20 weeks’ gestation) of the treatment; and the use of low dose aspirin in this subgroup could be beneficial, particularly if started before 16 weeks’ gestation [13].

Maternal age and parity had no significant effect on the risk of superimposed preeclampsia in our study. A similar lack of effect of maternal age in women with chronic hypertension was found by Sibai and colleagues [7]. This is in contrast to recent reports where nulliparity [14], and maternal age [15] were associated with increased risk of preeclampsia in unselected women. Therefore, it is not clear whether multiparity or lower maternal age are protective in pregnant women with essential chronic hypertension, highlighting if necessary that surveillance should be heightened for these women.

Duration of hypertension prior to pregnancy of more than 4 years has been associated with increased risk of superimposed preeclampsia [7]. Our study, however, did not confirm this finding, and is in agreement with a recent one [3].

The prevalence of black ethnicity (53.6%) in chronic hypertensive women is quite high and it does not reflect our general population. It is, however, important to note that ethnicity was not independently associated with superimposed preeclampsia in our study.

In several studies, pregnant women with essential chronic hypertension had high rates of fetal growth restriction (<5th birth weight percentile) without superimposed preeclampsia [5], [6]. In our study where 44 babies were <5th birth weight percentile, only 12 (20.7%) were observed in women having superimposed preeclampsia whereas the latter 32 (17.2%) were found in women without superimposed preeclampsia (non significant difference). These findings show that women with essential chronic hypertension are at risk for severe fetal growth restriction irrespective of the occurrence of superimposed preeclampsia. The explanation may be an overcorrection of blood pressure suggested by some authors [16], and in our study, treatment was stopped for 16 patients because of a SBP under 120 mmHg and DBP under 70 mmHg. A recent meta-analysis, however, did not find such relationship [17].

Our study has some limitations. The number of patients is relatively small. It is to note, however, that our population concerned exclusively women with essential chronic hypertension who had a treatment at first prenatal visit, and this is not the case of the majority of published studies. Another limitation is related to the retrospective structure of the study, for instance, we did not make any confirmation on past obstetric history that were mentioned in the medical charts, and this may induce some errors in the analysis [18]. In addition, an important number of women transferred from other centers (n = 61), and not followed in both maternities since the beginning of their pregnancy were excluded from the study because the management of antihypertensive treatment in these women could have been different. Moreover, almost all these patients had an adverse pregnancy outcome, and therefore the results would have been biased. Finally, gestational age at first prenatal visit is relatively late (18.1 wks). This result, however, reflects our clinical practice and it still remains possible to introduce some preventive treatment such as aspirin and to plan a close monitoring of the pregnancy.

### Conclusion

In essential chronic hypertensive women treated before the pregnancy, previous preeclampsia, and mean arterial blood pressure ≥95 mmHg are associated with increased risk of superimposed preeclampsia. Together, these two variables may select women at extreme high risk of superimposed preeclampsia.
